# Dental Shame: A Call for Understanding and Addressing the Role of Shame in Oral Health

**DOI:** 10.1111/cdoe.70019

**Published:** 2025-09-21

**Authors:** Louise Folker, Luna Dolezal, Astrid Pernille Jespersen, Martha Paisi, Lyndsey Withers, Christina Worle, Esben Boeskov Øzhayat

**Affiliations:** ^1^ Copenhagen Centre for Health Research in the Humanities (CoRe) Saxo Institute, Faculty of Humanities. South Campus. University of Copenhagen Copenhagen S Denmark; ^2^ Department of Odontology, Faculty of Health and Medical Sciences University of Copenhagen Copenhagen N Denmark; ^3^ Centre for Cultures and Environments of Health University of Exeter Exeter UK; ^4^ Peninsula Dental School University of Plymouth Plymouth Devon UK; ^5^ Dental Department HMP Eastwood Park Gloucestershire

**Keywords:** dental anxiety, dental shame, ohrool, oral health inequities, shame, stigma

## Abstract

This commentary highlights dental shame as a pervasive but underexplored phenomenon with significant implications for oral health and systemic inequities. It proposes dental shame as a critical lens for understanding and addressing the complex interplay between personal, social, economic, cultural and systemic factors contributing to oral health challenges. Drawing on interdisciplinary expertise, the authors collectively propose that dental shame is both a consequence and determinant of oral health issues, leading to a self‐reinforcing dynamic of avoidance, withdrawal and exacerbated inequities. They identify five key aspects of oral health where dental shame warrants deeper investigation: clinical encounters, care and social services, daily oral health behaviours, systemic inequities and psychological trauma. On this basis, the authors call for more research on dental shame and advocate for shame‐sensitive practices in dentistry and other health care and social settings. This approach includes fostering shame competence in practitioners, addressing systemic barriers and designing empathetic, inclusive care environments. Ultimately, the authors state that understanding and addressing dental shame can transform oral health promotion, reduce inequities and improve overall health outcomes.

## 1 Why Dental Shame?

Dental shame is an unilluminated yet widespread phenomenon, holding profound implications for oral health and oral health inequities. In this paper, we propose the concept of dental shame as a lens to understand the social, cultural, personal, economic and systemic aspects that are part and parcel of oral health. We state that examining the most significant challenges within dentistry—social inequities in oral health, stigma, insufficient daily oral care and lack of attachment to dental care systems [1] through the lens of shame can provide a deeper understanding of their complex and diverse dynamics, including their intertwining. We suggest that a deeper understanding of dental shame can alleviate some of the devastating consequences that oral health problems can have on overall health, disease and even death [1]. Further, we state that such understanding holds great potential in developing interventions to address these critical consequences, ultimately improving patient outcomes.

We are a diverse collective of professionals spanning various fields within and beyond academia, united by our commitment to shed light on dental shame. Our collective includes public oral health researchers from the Department of Odontology and cultural researchers from Copenhagen Centre for Health Research in the Humanities (CoRe), both at the University of Copenhagen, who work together on the project Lifelong Oral Health, which aims to identify barriers to oral health in Danish elderly care—and have identified dental shame as significant in elderly care settings [2]. Additionally, our alliance includes researchers affiliated with the Shame and Medicine research project at the University of Exeter in the UK, which uses interdisciplinary research to explore the role of shame in health and medicine. Concurrently, we benefit from the expertise of practising dentists and academics working with voluntary services serving marginalised communities. Drawing from our cross‐disciplinary backgrounds, we collectively call upon researchers, practitioners, educators, policymakers and funding bodies to recognise the importance of dental shame, as its recognition enables a comprehensive approach to oral health promotion and care.

## 2 State of the Art

We contend that the emotional complexities surrounding oral health issues, behaviours and inequities require a deeper understanding beyond the confines of existing odontological concepts; and we contend that shame is a beneficial focus in such an understanding. Even though some literature acknowledges the presence of shame in relation to oral health, it has not been extensively studied as an independent focus. Shame appears tangentially in research concerning Oral Health‐related Quality of Life (OHRQoL) [3, 4, 5, 6, 7, 8], dental anxiety [9, 10, 11, 12, 13, 14] and oral health‐related stigma [15, 16], all of which provide valuable insights. However, we posit that the intricate interplay between oral health and shame necessitates direct independent exploration, offering additional layers of understanding.

OHRQoL primarily examines the impact of oral disease on individuals' lives [17] but does not extend into addressing the underlying causes and social implications of such diseases. We suggest that delving into dental shame can illuminate these crucial aspects. Further, dental anxiety has garnered significant attention in understanding the avoidance of dental care. Shame provides a more precise explanation in cases where individuals are not necessarily anxious about treatment but rather about exposing their teeth, and relevant lifestyle factors (e.g., smoking, diet), for instance, to dental practitioners. Since anxiety often manifests as a response to shame, focusing solely on dental anxiety may obscure the underlying shame component, which is identified in shame research as *shame anxiety* [18, 19, 20]. Likewise, stigma and shame are often conflated, but they represent distinct concepts. While shame is directly emotionally experienced, stigma is not. Instead, stigma is a broader concept that refers to the social processes and judgments that mark certain individuals or groups as different or undesirable [19]. For instance, Doughty and colleagues have recently conceptualised oral health stigma as processes that “*include labelling, stereotyping, social exclusion, and discrimination enacted by society at large on individuals or groups with marked deviations from dominant oral health norms”* [15]. While it is clear that stigma may lead to shame, and the concepts are strongly entangled, it is important to note that shame and stigma cannot be conflated. Not all shame is related to stigma, and stigma does not encapsulate the personal experience of shame itself [19]. Therefore, shame emerges as a key concept for understanding first‐person lived experience and for enhancing oral health outcomes. Viewing oral health through the lens of shame thus offers added value to these three research areas with significant perspectives on the underlying causes of oral health issues and their consequences, paving the way for novel insights and solutions.

Shame arises when we are concerned about negative judgementfrom others or when we have transgressed a social norm or rule [21]. It is a complex array of various negative self‐conscious emotions, which have been described as “mercurial” [22], in so far as they are changeable and unpredictable. It includes painful emotions that can make us feel as though our social bonds are under threat or as though we might be rejected from our social group. We are reluctant to admit to shame or visibly show signs of experiencing shame because shame itself is taboo and also a source of shame [23]. Therefore, individuals will go to great lengths to avoid shame and shameful exposure. As a result, shame powerfully drives decision‐making and behaviour, even when this means hurting or disadvantaging oneself. Existing research from other parts of healthcare and medicine demonstrates that shame can interfere with everyday health behaviours, treatment‐seeking and adherence [19, 24, 25] and encounters within various healthcare settings and social services [26, 27], ultimately causing and exacerbating health issues and inequities [23]. Furthermore, shame tends to shape and define healthcare encounters, often leading to avoidance of what causes shame. Therefore, shame has been identified as a “potent treatment barrier” [27]. Furthermore, shame is intimately related to social power and strongly intersects with social categories such as gender, ethnicity, class and race [28, 29] and with conceptions of beauty [30]. This means that some people, especially in minoritised positions, may be disproportionately burdened by shame, highlighting shame's intertwining with power imbalances and inequities. Drawing on this literature, we suggest that similar dynamics exist regarding oral health.

Shame is situated and highly shaped by the personal and cultural contexts in which it appears [31]. While norms about what is 'acceptabl' and 'unacceptabl' or 'norma' and 'abnorma', and hence 'shameful' or 'not shameful', can vary between cultures, societies and even families, there is also variability within individuals regarding triggers for shame, and this might be dependent on personality traits, personal morals, religious beliefs, upbringing and trauma history, among other factors. On this basis, dental shame may arise differently in Denmark and the UK, depending on national standards of oral health, healthcare user fee structures and welfare services, as different systems create different kinds of inequities. Within these national differences, there may be significant variability at the individual level regarding when and how dental shame manifests, especially when considering health inequities. However, we recognise general elements of dental shame that transcend these differences in broader contexts.

## 3 What Is Dental Shame?

We define dental shame broadly and have identified three overall situations in which it tends to occur. First, dental shame can stem directly from oral health issues or the aesthetic appearance of the teeth, encompassing especially visible dental problems, such as broken, carious, darkened and missing teeth, as well as oral malodours and difficulties with the functioning of the teeth when eating, drinking and speaking. Second, dental shame can originate from broader social vulnerabilities, encompassing a more complex intertwining between shame and oral health. For instance, social and economic circumstances may markedly influence the degree to which someone can maintain and improve their oral health, appearance, and function [32]. This is further compounded by social vulnerabilities and deprivation, such as experiences of trauma [33] and abuse, food poverty [34], low literacy and harmful coping strategies such as substance abuse [35]. Yet, this does not simply apply to people in marginalised life situations, but also occurs more broadly in society, for instance, regarding eating, drinking or smoking habits or financial prioritisation. Third, dental shame is a relational phenomenon, meaning one can experience it on behalf of others, including children [36]. Dental shame can thus draw other people into sensitive shame situations, making people uncertain about how to address a person with oral health issues, potentially impacting relations, social life, work life, and encounters with dental practitioners and other healthcare professionals [2]. In each case, we suggest dental shame can lead to lower self‐worth, social isolation and unfavourable oral health care behaviours.

Drawing on our different professional approaches to shame and oral health, we have identified that dental shame is both a consequence and a determinant of oral health issues. It is a consequence because oral health issues can cause shame, and it is a determinant because it can act as a barrier to both daily dental care and engagement with dentistry. We suggest that this duality can turn dental shame into a self‐reinforcing spiral, where shame about oral health can lead to unfortunate oral health behaviours, which can potentially intensify oral health issues and inequities, leading to more shame. Furthermore, this dynamic has consequences beyond oral health. Because our teeth are highly visible and central to our overall appearance and well‐being, dental shame affects self‐esteem, social interactions, access to the labour market, care systems and social services [37, 38]. Thus, this downward spiral concerns not only oral health but also various other aspects of life.

## 4 The Shame Lens

Shame plays a dual role in our social life and health behaviours. On the one hand, it plays an essential pro‐social function, leading individuals to learn and grow, creating social harmony and motivating positive behaviour change [39] ‐ for instance, it can motivate positive changes in (oral) health behaviours. Therefore, shame is sometimes mobilised in public health campaigns [40]. However, it must be acknowledged that shame is also often a profoundly anti‐social emotion that can lead to distress. Moreover, healthcare practitioners can incite shame in patients both intentionally and unintentionally. When shaming is used purposefully with the intention to attempt to motivate positive health behaviours, there is no guarantee this will result in positive change; patients can experience this shaming as harmful [41]. We suggest that this dynamic is often at stake in dentistry and other (oral) health care encounters, necessitating enhanced knowledge about dental shame both in theory and in practice.

We call for the use of the shame lens within oral health, as it provides an important focus that will improve outcomes and make services more sensitive and humane [42]. Using the shame lens directs attention to how shame might be manifesting for individuals and the coping or defensive behaviours it might cause (e.g., avoidance, withdrawal, deflection and defensiveness), which may hitherto not have been a consideration within healthcare encounters. Adopting the shame lens also helps reveal how shame or shaming might be embedded within policies, practices or the material conditions of an organisation. It therefore offers systems and practitioners a chance to not reinforce or induce dental shame but to deal with it inclusively and empathically, creating non‐judgmental environments where patients feel trustful and empowered to prioritise their oral health.

We recognise various aspects of oral health that we believe would benefit from examination through the shame lens. We have identified five key aspects below that underscore the influence of dental shame as a critical factor in oral health. They can be deeply intertwined and overlapping but also address essentially different contexts, life circumstances and societal groups. It is important to acknowledge that they represent only some of the potential aspects in which dental shame may exert influence, suggesting the need for further investigation into its wider manifestations. The following outlines some of the prominent oral health aspects where the shame lens serves as a relevant focus for future research:

### 4.1 Dental Shame in Dentistry and Clinical Encounters

Examining shame from both a patient's and dental practitioner's perspectives reveals its pervasive presence within clinical settings. Patients may experience shame stemming from various sources, such as dental neglect, fear of judgment or socioeconomic factors influencing their oral health status. On the other hand, dentists encounter challenges in navigating the balance between providing effective care and mitigating shame‐induced barriers to communication and treatment participation. Understanding and addressing shame within these potentially precarious encounters are essential for achieving trust, initiating conversations, improving patient‐provider relationships and transforming everyday dental care habits.

### 4.2 Dental Shame in Healthcare and Social Services

Beyond the traditional clinical settings, dental shame permeates interactions with healthcare workers and social workers engaged in addressing (oral) health‐related challenges—for instance, professionals in psychiatry, elderly care, social pedagogy and employment agencies. Such settings are very relevant for people who are not connected to dental systems or who do not visit their dentist due, for example, to dental shame. Acknowledging and addressing dental shame in these contexts is crucial to creating supportive environments that contribute to equitable access to care without judgment or stigmatisation.

### 4.3 Dental Shame in Everyday Life and Oral Health Behaviours

The influence of shame extends beyond clinical and institutional encounters, being deeply intertwined with individuals' everyday life practices and behaviours pertaining to oral health. Exploring the entanglements between shame and everyday lifestyle factors, which may impact dental health (e.g., smoking, diet), in addition to everyday dental care habits, including dental neglect, offers important insights into the underlying psychosocial determinants behind oral health behaviours. By investigating the complex entanglements between dental shame, societal norms and individual agency, future research can develop knowledge and interventions aimed at transforming oral health behaviours in everyday life.

### 4.4 Dental Shame in Broader Systemic Contexts

Systemic inequities in dental care significantly contribute to dental shame and its complex and self‐reinforcing dynamics. Of significance are healthcare fee structures, which often increase dental shame and are thus a prominent factor. This can be the case for all people, including people who can afford and prioritise seeing a dentist. However, it especially rings true among marginalised populations. Understanding and addressing dental shame within a broader systemic context is thus essential for addressing oral health inequities. This further applies to access to dental care and differences in the acquisition of daily dental habits in childhood. A systemic approach to dental shame also includes addressing oral health issues related to homelessness, substance abuse and violence, for instance, domestic abuse, which already carries high levels of shame and stigma. By critically examining how oral health issues relate to these different forms of oppression and vulnerabilities through the shame lens, future research can advocate for structural interventions aimed at dismantling systemic barriers. Understanding the complex interplay between dental shame and social status is critical to developing policies, interventionsand campaigns promoting equitable access to dental care and mitigating harmful effects on dental health outcomes. Such interventions can potentially promote inclusive, accessible and culturally responsive oral health systems.

### 4.5 Dental Shame in Relation to Trauma

There is a great deal of evidence demonstrating that trauma and shame are closely linked [36] and that individuals who have experienced trauma are very likely to be more shame‐prone or more likely to be anticipating shame in interpersonal encounters. This can be exacerbated when coming into contact with professional services, like healthcare and social work, which have been identified to be potential “vectors of shame, humiliation and inequality” [43]. Hence, individuals who have experienced trauma, or are living in post‐trauma states, may be more likely to display shame defence behaviours such as avoidance. Understanding how these dynamics influence oral health behaviour and dentistry encounters is, therefore, potentially crucial.

## 5 Addressing Dental Shame

By designing practices and services to be “shame‐sensitive” [42], there are opportunities to mitigate the negative effects of dental shame. Shame‐sensitive practice involves using the “shame lens” to identify where the potential for shame may be embedded in organisational policy, practiceand education while also creating heightened sensitivity to the potential for shame to be present in clinical encounters and other interpersonal interactions within and beyond dentistry. To do so, practitioners in (oral) health care and social settings should be trained to have “shame competence” [44], which is a set of skills and knowledge that enables greater adeptness at identifying shame in oneself or others, being aware of how shame circulates between individuals and within institutional culture, managing shame dynamics, identifying shaming in policy and practice [43] and reducing the potentially damaging and anti‐social effects of shame [44]. Individual shame competence must be developed within a framework of organisational shame competence. This involves the fostering of emotional intelligence within workplaces and professional and community‐based practice, where speaking about and understanding emotions and their effects becomes normalised and commonplace. In particular, the taboo and stigma that surround shame, and shameful or stigmatised states and experiences, must be directly addressed and discussed openly.

## 6 Conclusion and Call for Further Research

With this position paper, we call for further research into the dynamics of dental shame. As we have underscored the breadth and multi‐faceted nature of dental shame across social and cultural divides, we emphasise the need for continued investigation to fully illuminate its implications. By integrating a shame lens into future dental research and practice, we identify a significant potential to advance both knowledge and social justice within the field, both regarding prevention and treatment. Moreover, we propose designing shame‐sensitive services in dentistry and beyond because of the enabling potential to address some of the most significant challenges in dentistry [1]. We further propose integrating shame competence in dentistry education and training to enable the acknowledgement and addressing of dental shame in practice, for instance, by involving people or communities with lived experience of dental shame. By creating a multidimensional understanding of dental shame and its implications for oral health, we thus suggest that future research can contribute to the development of more compassionate and effective approaches to oral health promotion.

## Conflicts of Interest

The authors declare no conflicts of interest.

## Data Availability

Data sharing not applicable to this article as no datasets were generated or analysed during the current study.

## References

[1] Global Oral Health Status Report: Towards Universal Health Coverage for Oral Health by 2030 (World Health Organization, 2022).

[2] L. Folker , A. P. Jespersen , and E. B. Øzhayat , “Tooth Shame ‐ an Ethnographic Study of the Choreographies of Tooth Shame in Danish Elderly,” Social Science & Medicine (2024): 117500. 39644775 10.1016/j.socscimed.2024.117500

[3] P. F. Allen , “Assessment of Oral Health Related Quality of Life,” Health and Quality of Life Outcomes 1 (2003): 40. 14514355 10.1186/1477-7525-1-40 PMC201012

[4] F. Allen and D. Locker , “A Modified Short Version of the Oral Health Impact Profile for Assessing Health‐Related Quality of Life in Edentulous Adults,” International Journal of Prosthodontics 15, no. 5 (2002): 446–450. 12375458

[5] A. S. Brisman , “Esthetics: A Comparison of Dentists' and Patients' Concepts,” Journal of the American Dental Association 100, no. 3 (1980): 345–352. 6928165 10.14219/jada.archive.1980.0093

[6] C. Derblom , M. L. Hagman‐Gustafsson , and P. Gabre , “Older People's Description of Factors That Facilitate and Impede Regular Dental Care – A Qualitative Interview Study,” International Journal of Dental Hygiene 15, no. 4 (2017): 313–320. 27868346 10.1111/idh.12263

[7] I. Gragoll , L. Schumann , M. Neubauer , C. Westphal , and H. Lang , “Healthcare Avoidance: A Qualitative Study of Dental Care Avoidance in Germany in Terms of Emergent Behaviours and Characteristics,” BMC Oral Health 21, no. 1 (2021): 563. 34743719 10.1186/s12903-021-01933-1 PMC8574006

[8] M. Mehrstedt , M. T. John , S. Tönnies , and W. Micheelis , “Oral Health‐Related Quality of Life in Patients With Dental Anxiety,” Community Dentistry and Oral Epidemiology 35, no. 5 (2007): 357–363. 17822484 10.1111/j.1600-0528.2007.00376.x

[9] E. Bryne , S. C. P. D. Hean , K. B. Evensen , and V. H. Bull , “Seeing the Person Before the Teeth: A Realist Evaluation of a Dental Anxiety Service in Norway,” European Journal of Oral Sciences 130, no. 3 (2022): e12860. 35218586 10.1111/eos.12860 PMC9306951

[10] H. Buchanan and N. S. Coulson , “Accessing Dental Anxiety Online Support Groups: An Exploratory Qualitative Study of Motives and Experiences,” Patient Education and Counseling 66, no. 3 (2007): 263–269. 17320336 10.1016/j.pec.2006.12.011

[11] S. M. Cohen , J. Fiske , and J. T. Newton , “The Impact of Dental Anxiety on Daily Living,” British Dental Journal 189, no. 7 (2000): 385–390. 11081950 10.1038/sj.bdj.4800777

[12] A. H. Schuurs , H. J. Duivenvoorden , P. C. Makkes , S. K. Thoden van Velzen , and F. Verhage , “Personality Traits of Patients Suffering Extreme Dental Anxiety,” Community Dentistry and Oral Epidemiology 16, no. 1 (1988): 38–41. 3422617 10.1111/j.1600-0528.1988.tb00552.x

[13] R. Moore , I. Brødsgaard , and N. Rosenberg , “The Contribution of Embarrassment to Phobic Dental Anxiety: A Qualitative Research Study,” BMC Psychiatry 4 (2004): 10. 15096278 10.1186/1471-244X-4-10 PMC411042

[14] J. M. Armfield , J. F. Stewart , and A. J. Spencer , “The Vicious Cycle of Dental Fear: Exploring the Interplay Between Oral Health, Service Utilization and Dental Fear,” BMC Oral Health 7 (2007): 1. 17222356 10.1186/1472-6831-7-1 PMC1784087

[15] J. Doughty , M. E. Macdonald , V. Muirhead , and R. Freeman , “Oral Health‐Related Stigma: Describing and Defining a Ubiquitous Phenomenon,” Community Dentistry and Oral Epidemiology 51 (2023): 1078–1083. 37462247 10.1111/cdoe.12893

[16] V. Muirhead , A. Milner , R. Freeman , J. Doughty , and M. E. Macdonald , “What Is Intersectionality and Why Is It Important in Oral Health Research?,” Community Dentistry and Oral Epidemiology 48 (2020): 464–470. 32840901 10.1111/cdoe.12573

[17] D. Locker , “Measuring Oral Health: A Conceptual Framework,” Community Dental Health 5, no. 1 (1988): 3–18. 3285972

[18] N. W. Brown , The Destructive Narcissistic Pattern (Praeger Publishers/Greenwood Publishing Group, 1998), 185.

[19] L. Dolezal , “Shame Anxiety, Stigma and Clinical Encounters,” Journal of Evaluation in Clinical Practice 28, no. 5 (2022): 854–860. 35903848 10.1111/jep.13744 PMC7613638

[20] H. B. Lewis , “Shame and Guilt in Neurosis,” Psychoanalytic Review 58 (1971): 419–438. 5150685

[21] D. L. Nathanson , Shame and Pride: Affect, Sex, and the Birth of the Self (W. W. Norton & Company, 1994), 500.

[22] E. K. Sedgwick and F. Adam , “Shame in the Cybernetic Fold: Reading Silvan Tomkins,” Critical Inquiry 21, no. 2 (1995): 496–522.

[23] L. Dolezal and B. Lyons , “Health‐Related Shame: An Affective Determinant of Health?,” Medical Humanities 43, no. 4 (2017): 257–263. 28596218 10.1136/medhum-2017-011186 PMC5739839

[24] “Stage C. Shame, Chronic Illness and Participatory Storytelling,” Body & Society 28, no. 4 (2022): 3–27.

[25] P. Gilbert and J. Miles , Body Shame: Conceptualisation, Research, and Treatment (Psychology Press, 2002), 328.

[26] F. Davidoff , “Shame: The Elephant in the Room,” BMJ 324, no. 7338 (2002): 623–624. 11895807 10.1136/bmj.324.7338.623 PMC1122563

[27] T. Saraiya and T. Lopez‐Castro , “Ashamed and Afraid: A Scoping Review of the Role of Shame in Post‐Traumatic Stress Disorder (PTSD),” Journal of Clinical Medicine 5, no. 11 (2016): 94. 27809274 10.3390/jcm5110094 PMC5126791

[28] S. E. Raskin , M. Thakkar‐Samtani , M. Santoro , E. B. Fleming , L. J. Heaton , and E. P. Tranby , “Discrimination and Dignity Experiences in Prior Oral Care Visits Predict Racialized Oral Health Inequities Among Nationally Representative US Adults,” Journal of Racial and Ethnic Health Disparities 11, no. 6 (2024): 3722–3735. 37848669 10.1007/s40615-023-01821-0 PMC11564283

[29] S. Raskin , Stigma and Synergies of Dental Disease, Diabetes, and Psychosocial Stress Among Low‐Income Rural Appalachians, vol. 7 (Stigma Syndemics: New Directions in Biosocial Health, 2017), 193.

[30] A. Khalid and C. Quiñonez , “Straight, White Teeth as a Social Prerogative,” Sociology of Health & Illness 37, no. 5 (2015): 782–796. 25923766 10.1111/1467-9566.12238

[31] C. Mun , Cultural Perspectives on Shame: Unities and Diversities (Routledge, 2023), 244.

[32] N. Su , D. Duijster , G. J. M. G. van der Heijden , J. O. Groeniger , and M. A. Beenackers , “The Role of Psychological Distress in the Relationship of Financial Strain With Oral Health and Dental Attendance in Dutch Adults: A Mediation Analysis Based on Cross‐Sectional Data,” Community Dentistry and Oral Epidemiology 52, no. 5 (2024): 749–758. 38750647 10.1111/cdoe.12974

[33] A. A. Akinkugbe , V. Midya , M. A. Crane , D. T. Garcia , U. S. Clark , and R. J. Wright , “Long‐Term Oral Health Effects of Traumatic Events Among World Trade Center Health Registry Enrolees, 2003–2020,” Community Dentistry and Oral Epidemiology 53 (2025): 170–179. 39582260 10.1111/cdoe.13020 PMC11893248

[34] G. Carbajal Rodriguez , A. W. van Meijeren‐van Lunteren , E. B. Wolvius , and L. Kragt , “Poverty Dynamics and Caries Status in Young Adolescents,” Community Dentistry and Oral Epidemiology 53, no. 1 (2024): 90–97. 39390670 10.1111/cdoe.13012 PMC11754150

[35] S. Wickholm , M. R. Galanti , B. Söder , and H. Gilljam , “Cigarette Smoking, Snuff Use and Alcohol Drinking: Coexisting Risk Behaviours for Oral Health in Young Males,” Community Dentistry and Oral Epidemiology 31, no. 4 (2003): 269–274. 12846849 10.1034/j.1600-0528.2003.00046.x

[36] T. S. Carvalho , J. Abanto , E. C. M. Pinheiro , A. Lussi , and M. Bönecker , “Early Childhood Caries and Psychological Perceptions on Child's Oral Health Increase the Feeling of Guilt in Parents: An Epidemiological Survey,” International Journal of Paediatric Dentistry 28, no. 1 (2018): 23–32. 28514517 10.1111/ipd.12306

[37] E. B. Øzhayat , S. Åkerman , N. Lundegren , and B. Öwall , “Patients' Experience of Partial Tooth Loss and Expectations to Treatment: A Qualitative Study in Danish and Swedish Patients,” Journal of Oral Rehabilitation 43, no. 3 (2016): 180–189. 26426127 10.1111/joor.12355

[38] A. M. Sigsgaard , I. Bolvig , K. D. Jensen , S. Altmann , B. Hede , and E. B. Øzhayat , “Oral Health Promotion and Labour Market Prospects of Socially Disadvantaged and Unemployed People ‐ a Randomised Controlled Trial,” Scandinavian Journal of Public Health 52, no. 1 (2024): 71–79. 35510343 10.1177/14034948221092577 PMC11328448

[39] C. Sanderson , Counselling Skills for Working With Shame (Jessica Kingsley Publishers, 2015).

[40] A. Brewis and A. Wutich , Lazy, Crazy, and Disgusting (Johns Hopkins University Press, 2022).

[41] F. Cooper , L. Dolezal , and A. Rose , “Shame‐Sensitive Public Health,” Journal of Medical Humanities 46 (2024): 59–73. 39042177 10.1007/s10912-024-09877-7 PMC7616610

[42] L. Dolezal and M. Gibson , “Beyond a Trauma‐Informed Approach and Towards Shame‐Sensitive Practice,” Humanities and Social Sciences Communications 9, no. 1 (2022): 1–10. 10.1057/s41599-022-01227-z PMC7612965 35791341

[43] M. Salter and H. Hall , “Reducing Shame, Promoting Dignity: A Model for the Primary Prevention of Complex Post‐Traumatic Stress Disorder,” Trauma, Violence, & Abuse 23, no. 3 (2022): 906–919. 10.1177/1524838020979667 33345743

[44] L. Dolezal and W. Bynum , “Shame Competence: Addressing the Effects of Shame in Health Care,” Lancet 404, no. 10462 (2024): 1514–1515. 39426826 10.1016/S0140-6736(24)02269-4

